# How does functionality proceed in ACL reconstructed subjects? Proceeding of functional performance from pre- to six months post-ACL reconstruction

**DOI:** 10.1371/journal.pone.0178430

**Published:** 2017-05-31

**Authors:** Frieder Cornelius Krafft, Bernd Josef Stetter, Thorsten Stein, Andree Ellermann, Johannes Flechtenmacher, Christian Eberle, Stefan Sell, Wolfgang Potthast

**Affiliations:** 1 Sports Orthopaedics, Institute of Sports and Sports Science, Karlsruhe Institute of Technology (KIT), Karlsruhe, Germany; 2 BioMotion Center, Institute of Sports and Sports Science, Karlsruhe Institute of Technology (KIT), Karlsruhe, Germany; 3 Arcus Sports Clinic, Pforzheim, Germany; 4 Ortho-Zentrum, Karlsruhe, Germany; 5 Hospital Neuenbürg, Joint Center Black Forest, Neuenbürg, Germany; 6 Institute for Biomechanics and Orthopaedics, German Sport University (GSU), Cologne, Germany; Nanyang Technological University, SINGAPORE

## Abstract

This is the first study examining functionality of subjects with anterior cruciate ligament (ACL) tears and a subsequent reconstruction comprehensively by multiple test sessions from pre- to six months post-reconstruction. The purpose was to evaluate if a generally applied rehabilitation program restores functionality to levels of healthy controls. Subjects with unilateral tears of the ACL were compared to matched healthy controls throughout the rehabilitation. 20 recreational athletes were tested: T_1_ (preoperative), 6 weeks after tear; T_2_, 6 weeks, T_3_, 3 months, T_4_, 6 months post-reconstruction. At all test sessions, subjects self-evaluated their activity level with the Tegner activity score and their knee state with the Knee Injury and Osteoarthritis Outcome Score. Passive range of motion during knee flexion and extension and leg circumference were measured as functional clinical tests. Bilateral countermovement jumps, one-leg jumps for distance and isometric force tests in knee flexion and extension with 90° and 110° knee angle were conducted as functional performance tests. For determination of functionality, leg symmetry indices (LSIs) were calculated by dividing values of the injured by the uninjured leg. In the ACL group most LSIs decreased from T_1_ to T_2_, and increased from T_2_ and T_3_ to T_4_. LSIs of ACL subjects remained lower than LSIs of healthy controls at 6 months post-reconstruction in nearly all parameters. Self-evaluation of ACL subjects showed, additionally, that activity level was lower than the pre-injury level at 6 months post-reconstruction. Low LSIs and low self-evaluation indicate that knee joint functionality is not completely restored at 6 months post-reconstruction. The study shows that multiple comprehensive testing throughout the rehabilitation gives detailed images of the functional state. Therefore, the functional state of ACL reconstructed individuals should be evaluated comprehensively and continuously throughout the rehabilitation to detect persisting deficiencies detailed and adapt rehabilitation programs individually depending on the functionality.

## Introduction

Tears of the anterior cruciate ligament (ACL) can lead to chronic knee instability and a loss of joint function [1–3]. Common treatment of the torn ligament in industrial countries – e.g. Germany and USA [4, 5] – is the surgical reconstruction of the torn ligament. After the reconstruction a long-term rehabilitation process is required, which, however, does not ensure full stability and functionality of the knee joint in activities of daily living (ADL) and in sports activities. Thus, ACL ruptures, can highly influence the quality of life (QoL) and the subsequent ability to engage in sports on pre-injury level [1, 2, 5–9].

ACL tears lead to thigh muscle atrophy [10, 11]. Thigh muscle atrophy contributes to joint instability, because the muscles and ligaments surrounding the knee are crucial for knee stability and functionality during sports activities [2, 7, 12] and for maintaining stability and compensation of unexpected situations or postural balance disturbances in ADL [11, 13, 14]. Additionally, the sensory feedback from the mechanoreceptors of the torn ACL is deficient, which besides alters joint and locomotion biomechanics and therewith contributes instability processes [9, 14].

Studies of the last three decades show that the development of knee joint instabilities are multifactorial and therefore, no consensus about the origin and persistence of instabilities in elite and recreational athletes could be achieved [2, 3, 5, 15–28]. Due to the ACL tear, the injured leg as well as the uninjured leg can get influenced, resulting in a pathologic asymmetry level between the legs [16, 29]. However, it seems that task-specific symmetry levels in static and dynamic situations exist. Furthermore, symmetry levels are essential for full recovery of knee joint functionality and a safe return in ADL and sports activities [6, 31–33]. In order to quantify the symmetry level as a measure of knee joint functionality, the leg symmetry index (LSIs) is an established method [6, 16, 30–32]. To date no study investigated detailed functional characteristics of ACL reconstructed subjects longitudinally up to six months post-reconstruction by combining functional clinical tests, functional performance tests (FPTs) and questionnaires for functional self-evaluation. However, in long-term knee rehabilitation it is helpful to measure deficits of functionality repetitively from various viewpoints in order to develop more individualized rehabilitation programs for a high functional outcome. Furthermore, objective parameters determining functionality should be monitored and taken into consideration before ACL reconstructed individuals get released in pre-injury sports. Hence, it is necessary to understand how the specific biomechanical components, determining and limiting knee function (i.e. passive range of motion (ROM), muscular and neuromuscular capabilities in dynamic and static conditions), develop during the recuperation process after ACL reconstruction [5, 14, 17, 19, 31]. This is underlined by the results of various authors, which suggest a comprehensive assessment of functionality after ACL reconstruction from various viewpoints, instead of one specific viewpoint (i.e. the combination of different types of one-legged jumps (OLJs)) [7, 17, 19, 22, 24, 26, 27, 29, 32–35]. Such comprehensive assessments provide a broader picture of the knee joint functionality and can therefore help to gauge functional deficits more accurate. Accordingly, comprehensive studies should combine objective measures for both, clinical outcome and functional knee performance, along with functional self-evaluation of the ACL reconstructed subjects. With functional clinical tests (e.g. measurements of the knee’s passive ROM) the functionality of the knee is assessed under passive conditions [36, 37]. By functional performance tests (e.g. OLJs), the functionality of the knee joint is measured under specific dynamic conditions [15]. Thereby, the subjects need to generate active motor commands based on sensory information about the state of their body and the environment to coordinate the movements. Complementary, by self-evaluative questionnaires the subjects’ self-reflection about the knee functionality is assessed, which provides individual, examiner independent data from the subject’s point of view [38].

Therefore, the purpose of this study was to examine the functional state of ACL reconstructed subjects comprehensively by the combination of self-evaluating questionnaires, functional clinical as well as static and dynamic FPTs and in comparison to matched healthy control subjects. The implementation of such a test battery, along with a close monitoring of four test sessions up to six months post-reconstruction, will enable a more detailed understanding of the functional development of the knee status during rehabilitation. Therewith, a fine-grained picture of the subjects’ functional state at a specific time in the rehabilitation cycle can be provided. Such information can help clinicians and therapists to determine the functional knee state more comprehensively and to obtain more accurate criteria for decision making during the rehabilitation process [3, 5, 17, 19, 22, 23, 26, 33]. As ACL tears and reconstructions highly impact knee function, we hypothesized that in the post-reconstruction phase, subjects will gradually regain task-specific LSIs during the rehabilitation phase but will not reach the LSIs of healthy control subjects up to six months post-reconstruction.

## Methods

### Sample

Subjects with tears of the ACL (n = 20) and healthy control subjects (n = 20), without any history of leg injuries, participated in the study (Table 1). Inclusion criteria was that the subjects had unilateral tears and underwent uniform ACL reconstruction technique with a combined semitendinosus and gracilis autograft, via the single-bundle technique. Exclusion criteria were concomitant severe injuries of the Menisci or the collateral ligaments of the knee joint. Inclusion criteria of the control subjects was that they did not had any history of leg injuries. Control subjects were excluded if they had any leg injuries and if they did not fulfill the matching criteria. The control subjects were matched to the ACL subjects according to: sex, age, height, body mass and activity level before the ACL tear, as determined using the Tegner activity score (TAS). The study was approved by the ethics committee of the State Medical Council of Baden-Württemberg (Stuttgart, Germany). All subjects provided written informed consent for their study participation.

**Table 1:** Sample characteristics. Means and standard deviations.

	Age (yr)	Height (cm)	Mass (kg)	Body-mass index (kg/m^2^)	Activity Level (TAS)
**ACL group**	32.0 ± 13.3	174.7 ± 9.0	73.2 ± 8.7	24.1 ± 3.4	6.4 ± 1.4
**Control group**	33.3 ± 13.4	175.4 ± 10.4	74.7 ± 8.2	24.4 ± 2.6	6.0 ± 1.4

Mean values and standard deviations (SD) of the ACL subjects and the control subjects. ACL, anterior cruciate ligament; TAS, Tegner activity score; TAS in the ACL group subjects is related to the pre-injury activity level.

### Study design

**Fig 1:**
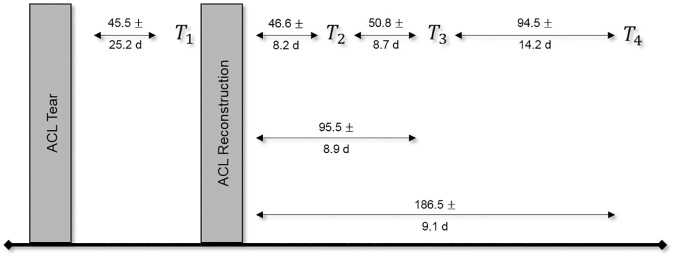
Study design. Showing the four conducted test sessions with time periods (Means and standard deviations in days [d]) between the respective test sessions.

As indicated in the introduction, a comprehensive understanding of the development of different components of knee function after ACL reconstruction is missing. Therefore, the study was designed as a longitudinal non-randomized controlled trial to evaluate an existing and commonly applied rehabilitation program after ACL reconstruction in a chronologically and functionality detailed manner. Therewith, it is assumable to identify possible time effects between or within parameters determining knee function and in comparison with healthy subjects. Accordingly, the ACL reconstructed subjects were tested at four different test sessions over a period of seven to eight months. The first test was performed preoperatively, immediately before the reconstruction and about seven weeks after the ACL tear (T_1_). All following tests were postoperative (T_2_-T_4_). T_2_ was about seven weeks, T_3_ was approximately three months and T_4_ approximately six months after ACL reconstruction. The control subjects attended only one test session. The test design was aligned to the three main stages of the rehabilitation process (Fig 1).

### Test battery

In the conducted test battery questionnaires for self-evaluation of the knee function, functional clinical tests and FPTs were combined. The selection of the tests should give a comprehensive image of the knee function and enables also good feasibility for practical implementations.

#### Questionnaires

We included questionnaires for self-evaluation of the knee function and the activity level in the test battery to receive independent data of the subjects’ view about the influence of the ACL injury to their general life. All subjects completed two questionnaires: The Knee Injury and Osteoarthritis Outcome Score (KOOS), for self-evaluation of the subjects’ knee function [38]. The KOOS consists of the sub-categories *Pain*, *Symptoms*, *Activities of daily living*, *Sport and recreation function*, and *Knee-related quality of life*. The whole questionnaire as all sub-categories are standardized to maximum reachable score of 100 [38]. For assessment of the subjects’ pre-injury and current activity levels, the TAS was applied [39].

#### Functional clinical tests

In addition to the questionnaires we included functional clinical tests in the test battery to measure the subjects’ knee functionality under static conditions. As functional clinical tests, leg circumference (LC) and passive ROMs of the knee joint were assessed. The LC was measured at four specific landmarks [36]: the joint line (JL), and 5 cm (S5) and 15 cm (S15) superior and 5 cm inferior (I5) to the joint line. The passive ROM of the knee joint was assessed three times during flexion prone and extension supine [37]. All ROM measurements were conducted by the examiner with a manual goniometer. The measurements were conducted at each leg separately to calculate the LSIs. Means of the three measurements were calculated for further analyses and for calculation of the LSIs.

#### Functional performance tests

Finally, we included FPTs, wherein subjects in contrast to the functional clinical tests need to actively generate motor commands to coordinate their movements. Subjects performed three countermovement jumps (CMJs) akimbo. The highest jump was used for analysis [27, 35]. While performing the CMJs, the subjects stood with each leg on a separate force plate (AMTI, 1000 Hz). Jumping height (absolute value), acceleration impulse during take-off (LSI) and the deceleration impulse during landing (LSI) were analyzed. Additionally, the subjects performed three one leg jumps (OLJs) for distance akimbo, with each leg. The subjects had to jump off and land on the same leg. Landing had to be stable with no movement of the landing foot and no ground contact of the contralateral leg. Landing pose had to be maintained for 3s. Jumps with the largest distance were used for LSI calculations of the jumping distances and acceleration impulses during take-off. Both jumping tests were applied to compare the functional state of the ACL reconstructed subjects in a one-legged and a bilateral movement.

The static muscular capabilities of knee flexion and knee extension musculature were measured under isometric conditions with a custom-made adjustable dynamometer rigid chair, equipped with a strain-gauge system (linear range, 0–2000 N; 1000 Hz; sensitivity, 3.6 mV/N). Isometric force tests were applied to get isolated information of the capabilities of the knee flexion and extension musculature. Isometric strength testing was applied because the reliability of isokinetic testing is reduced over higher ROMs, which is caused by the shift of the joint axes of the dynamometer in relation to the anatomical joint axes in isokinetic testing [40]. The muscular capabilities of both legs were assessed, in flexion and extension with knee angles of 90° and 110° (0° indicated a straight leg) [41]. The subjects were seated with a hip flexion angle of 90°. The tested leg was fixed in position with a strap around the malleoli. For each knee angle and type of contraction, two maximum voluntary contractions with 1-min rest periods were performed in a block-randomized order. The subjects were asked to produce their maximal force as fast as possible and to maintain the contraction between 3–5 s. The subjects received standardized verbal encouragement throughout every trial. To minimize extraneous body movements, straps were applied firmly across the shoulders, chest and stomach. Additionally, the subjects had to cross their arms over their chest to avoid any contribution of the trunk in force generation. The recorded signal was filtered through a digital fourth-order low-pass Butterworth filter, by using a cutoff frequency of 10 Hz. The trial with the highest maximum force was used for further analysis. Maximum force (F_max_), maximum rate of force development (RFD_max_) and RFD in 0–200 ms (RFD_200max_) were determined, and the LSIs for each of these parameters were calculated [13].

### Rehabilitation program

All subjects received a standardized post-surgical rehabilitation program, according to the German health insurance system. This consists of three stages: The first stage consists of low-intensity (passive) activities up to six weeks post-reconstruction. Including physiotherapy with lymphatic drainage, passive movement exercises (by machine or therapist), sensorimotor training, weight-bearing exercises and isometric training under therapists’ supervision. The second stage consists of medium-intensity activities with muscular and balance training up to three months post-reconstruction. Including physiotherapy with lymphatic drainage, passive movement exercises, independent strength training, balance training, and activities and sports not involving pivoting movements (e.g. cycling, swimming, (nordic) walking). The third stage consists of medium-to-high-intensity activities. Including intense strength training, if possible, up to six months post-reconstruction. As well, sports training (without pivoting movements) and slight return to pre-injury sports and sports-level with jumps, intense cycling and strength training. All stages were adaptable according to the rehabilitation state of the individuals’ knee joint. Such a stepwise, 3-staged structure is common in rehabilitation after ACL reconstruction [42]. The summarized rehabilitation program of the ACL subjects, including the applied exercises and training as well as the performable activities and sports, is presented in S1 Table.

### Data analysis

LSIs were calculated for all parameters by the related discrete values of the injured leg divided by the uninjured leg in the ACL subjects and by the non-dominant leg divided by the dominant leg in the control subjects, respectively. LSIs provide comparable results between all subjects. An LSI of 1.0 indicates that the performance of both legs was equivalent. LSIs are a widely used method to compare results between the legs and for determining functionality [2, 5, 6, 15–20, 23, 24, 26, 27, 31, 35, 43].

#### Statistics

Firstly, with Microsoft Office Excel 2013 means and 95% confidence intervals were calculated for the results of the questionnaires, for the LSIs of the functional clinical tests, and the LSIs and absolute values (jumping height in CMJs) of the FPTs. Afterwards, calculations for statistical interferences were conducted with IBM SPSS 22 (IBM, Armonk, NY, USA). First, Kolmogorov-Smirnov, and Mauchly’s tests were used to confirm the normality and sphericity of the data distribution. Greenhouse-Geiser estimates were used to correct for violations of sphericity.

Variations in the analyzed parameters for the ACL group over time (T_1_–T_4_) were assessed using one-way analysis of variance with repeated measures (RM-ANOVA). If the RM-ANOVA revealed a significant variation, the Holm-Bonferroni corrected post-hoc *t*-test for dependent samples was employed to determine statistical differences between the four test sessions [44]. Data of T_4_ in the ACL group were compared to the results of the control group, by using a *t*-test for independent samples in order to identify differences between control subjects and ACL subjects six months post-reconstruction. Effect sizes were calculated using partial eta squared for the RM-ANOVAs ($\eta_{p}^{2}$) and Cohen’s *d* for the *t*-tests. According to Cohen [45], large effects are indicated by $\eta_{p}^{2}$ = 0.14, medium-sized effects by $\eta_{p}^{2}$ = 0.06, and small effects by $\eta_{p}^{2}$ = 0.01. In terms of Cohen’s *d*, large effects are indicated by *d*=0.8, medium-sized effects by *d*=0.5 and small effects by *d*=0.2. The level of significance for all calculations was set a priori at *P≤*0.05.

## Results

### Questionnaires

#### KOOS questionnaire

The KOOS questionnaire was applied to examine the functional knee state from various viewpoints (symptoms & stiffness, pain, ADL, sports and recreational activities, and QoL) from the subjects’ self-evaluative view.

RM-ANOVA revealed a significant variation in symptoms & stiffness (*F*_(3,51)_=8.90, *P*<0.01, $\eta_{p}^{2}$ = 0.34), pain (*F*_(3,51)_=8.60, *P*<0.01, $\eta_{p}^{2}$ = 0.34), ADL (*F*_(3,51)_=7.39, *P*<0.01, $\eta_{p}^{2}$ = 0.30), sports and recreational activities (*F*_(3,51)_=20.86, *P*<0.01, $\eta_{p}^{2}$ = 0.55) and QoL (*F*_(3,51)_=14.13, *P*<0.01, $\eta_{p}^{2}$ = 0.45). Post-hoc analysis revealed significantly lower scores at T_2_ than at T_3_ in all subcategories. The ACL subjects had significantly lower scores at T_4_ than the control subjects in all subcategories. (Table 2)

**Table 2:** Mean results and standard deviations of the Knee Injury and Osteoarthritis Outcome scores’ subcategories.

Subcategory	T_1_	T_2_	T_3_	T_4_	Control group	Significant differences
**Symptoms & stiffness**	60.9 ± 19.9	55.0 ± 19.8	70.7 ± 15.0	74.3 ± 18.7	94.8 ± 8.1	T_2_/T_3_: *T*(17)=1.25, *P*=0.01, *d*=0.92T_4_/CG: *T*(38)=4.40, *P*<0.01, *d*=1.39
**Pain**	73.3 ± 13.3	70.6 ± 10.9	83.0 ± 7.6	84.1 ± 14.1	98.7 ± 3.7	T_2_/T_3_: *T*(17)=5.88, *P*<0.01, *d*=1.08T_4_/CG: *T*(38)=4.39, *P*<0.01, *d*=1.39
**ADL**	79.4 ± 16.5	78.1 ± 16.6	88.4± 16.0	91.4 ± 10.9	100 ± 0.0	T_2_/T_3_: *T*(17)=3.55, *P*<0.01, *d*=0.72T_4_/CG: *T*(38)=3.46, *P*<0.01, *d*=1.09
**Sports and recreational activities**	41.0 ± 18.2	36.3 ± 23.1	60.4 ± 24.4	69.0 ± 24.0	99.5 ± 1.5	T_2_/T_3_: *T*(17)=6.45, *P*<0.01, *d*=1.06T_4_/CG: *T*(38)=5.53, *P*<0.01, *d*=1.84
**QoL**	38.5 ± 15.5	40.3 ± 21.5	56.3 ± 22.8	59.6 ± 22.1	97.8 ± 2.6	T_2_/T_3_: *T*(17)=5.85, *P*<0.01, *d*=0.79T_4_/CG: *T*(38)=7.31, *P*<0.01, *d*=2.31

Results (means and standard deviations of all subjects) of the subcategories of the KOOS questionnaire of the ACL subjects (T_1_–T_4_) and the control group (CG). The subcategories are “symptoms & stiffness” (7 items), “pain” (9 items), “activities of daily living” (ADL; 17 items), “sports and recreational activities” (5 items), and “quality of life related to the knee injury” (QoL; 4 items). The maximum possible score in the KOOS was 100, indicating no symptoms. Significant differences (*P*≤0.05) with Cohen’s *d* between test sessions are illustrated in the last column.

Summarized, the ACL subjects evaluated their knee function higher at three months compared to six weeks after reconstruction. However, up to six months no further increase of the score was determined and it remained lower than the healthy control groups’ score.

#### TAS questionnaire

**Fig 2:**
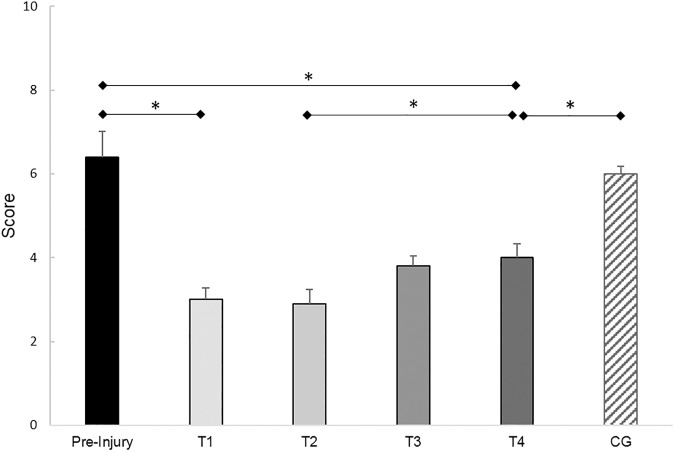
Results of the Tegner activity score. Mean activity level and 95% confidence intervals of the ACL subjects (T_1_–T_4_) and the control subjects, assessed with the Tegner activity score [37]. Test sessions with significant (*P*≤0.05) differences are marked with an asterisk (*).

RM-ANOVA revealed a significant variation in the TAS (*F*_(4,76)_=48.87, *P*<0.01, $\eta_{p}^{2}$ = 0.72). The ACL subjects had a significantly lower activity level at T_1_ than before the tear (*T*(19)=10.13, *P*<0.01, *d*=3.17). After reconstruction (T_2_), the activity level increased significantly up to T_4_ (*T*(19)=4.47, *P*<0.01, *d*=1.36). At T_4_, the activity level was still significantly lower than the pre-injury activity level (*T*(19)=8.72, *P*<0.01, *d*=2.01) and the activity level of the control subjects (*T*(38)=5.71, *P*<0.01, *d*=1.81) (Fig 2).

### Functional clinical tests

#### Leg circumference

**Fig 3:**
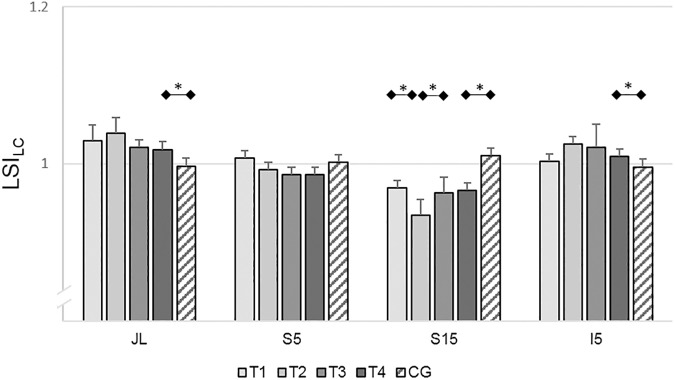
Results of the leg symmetry indices (LSIs) of leg circumference measurements. Mean LSIs and 95% confidence intervals of leg circumference measurements of the ACL subjects (T_1_-T_4_) and the control subjects. All subjects stood upright during the measurements. The circumference of the leg was measured at the joint line (JL), and 5cm (S5) and 15cm (S15) superior and 5cm inferior (I5) to the joint line [38]. Test sessions with significant (*P*≤0.05) differences are marked with an asterisk (*).

RM-ANOVA only revealed a significant variation in the LSI_LC_ at S15 (LSI_LCS15_) (*F*_(3,51)_=8.42, *P*<0.01, $\eta_{p}^{2}$ = 0.33). The ACL subjects had significantly lower LSIs_LCS15_ at T_2_ than at T_1_ (*T*(19)=4.53, *P*<0.01, *d*=1.02) and significantly higher LSI_LCS15_ at T_3_ than at T_2_ (*T*(17)=4.73, *P*<0.01, *d*=0.69). At all other landmarks (JL, S5, I5), no significant variations in LC could be found. In addition, the ACL subjects had significantly higher LSI_LC_ values at JL (*T*(38)=2.29, *P*=0.03, *d*=0.73) and I5 (*T*(38)=2.21, *P*=0.03, *d*=0.70) and significantly lower LSI_LC_ at S15 (*T*(38)=6.07, *P*<0.01, *d*=1.92) at T_4_ than the control subjects. No differences were detected at S5 between the ACL subjects at T_4_ and the control subjects (Fig 3).

Summarized, at six months post-reconstruction the knee joint area of the reconstructed leg is still thicker compared to the uninjured knee joint and in the middle of the thigh the circumference of the reconstructed leg is clearly reduced compared to the uninjured leg.

#### Passive ROM

**Fig 4:**
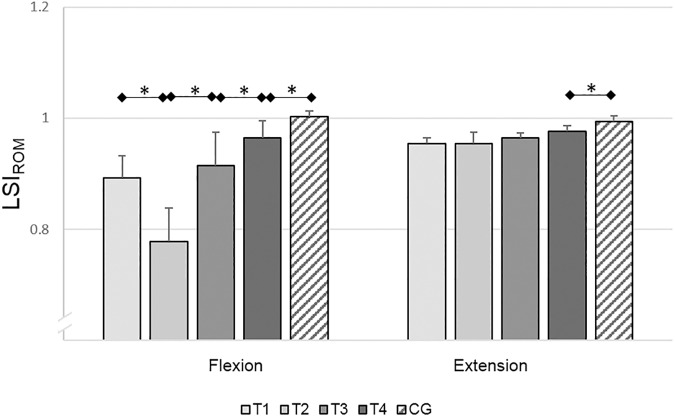
Results of the leg symmetry indices (LSIs) of the range of motion measurements. Mean LSIs and 95% confidence intervals of the range of motion (ROM) measurements. ROM was measured during knee flexion in prone position and knee extension in supine position in the ACL subjects (T_1_-T_4_) and the control subjects (CG) [39]. Test sessions with significant (*P*≤0.05) differences are marked with an asterisk (*).

RM-ANOVA revealed a significant variation for knee flexion (*F*_(3,51)_=31.65, *P*<0.01, $\eta_{p}^{2}$ = 0.65) but no variations for knee extension (*F*_(3,51)_=3.19, *P*=0.05, $\eta_{p}^{2}$ = 0.16). Post-hoc analysis showed that during knee flexion, the LSI_ROM_ was significantly lower at T_2_ than at T_1_ (*T*(19)=4.59, *P*<0.01, *d*=0.99), and significantly higher at T_3_ than at T_2_ (*T*(17)=7.39, *P*<0.01, *d*=1.20) and at T_4_ than at T_3_ (*T*(17)=3.75, *P<*0.01, *d*=0.69). In the ACL subjects at T_4_, the LSI_ROM_ during flexion (*T*(38)=3.89, *P*<0.01, *d*=1.23) and during extension (*T*(38)=2.65, *P<*0.01, *d*=0.84) was significantly lower compared to the control subjects. At T_4_, the deficit in the passive ROM of the injured legs was 3.5% in flexion and 2.3% in extension, compared to the uninjured leg (Fig 4). Regarding the passive ROM results, it is apparent that in knee flexion the ROM increases from six weeks post-reconstruction up to six months post-reconstruction. However, the side-to-side deficit in ACL reconstructed subjects remains significant compared to the healthy control subjects at six months post-reconstruction.

### Functional performance tests

#### Counter movement jumps (CMJ)

**Fig 5:**
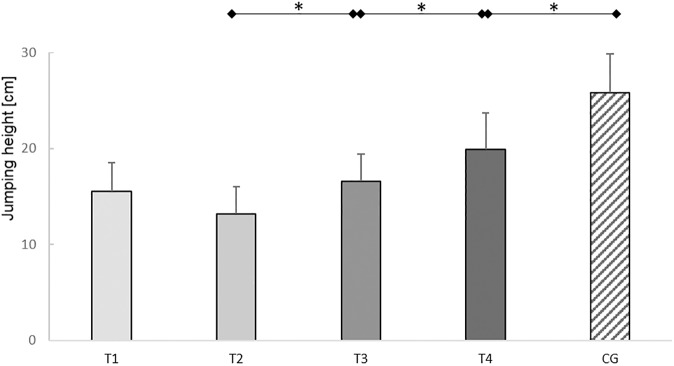
Results counter movement jumps (CMJs). Mean jumping heights and 95% confidence intervals of the ACL subjects (T_1_-T_4_) and control subjects (CG) of the CMJs. Test sessions with significant (*P*≤0.05) differences are marked with an asterisk (*).

RM-ANOVA revealed a significant variation for jumping heights (*F*_(3,33)_=5.88, *P*=0.01, $\eta_{p}^{2}$ = 0.35). Jumping heights were significantly higher at T_3_ than at T_2_ (*T*(11)=2.25, *P*=0.04, *d*=0.73) and at T_4_ than at T_3_ (*T*(17)=2.77, *P*=0.01, *d*=0.35). The jumping heights were significantly higher in the control subjects than in the ACL subjects at T_4_ (*T*(38)=2.08, *P*=0.04, *d*=0.66). In the ACL subjects, jumping heights increased by 50.8% from T_2_ to T_4_. The deficit in jumping heights in the ACL subjects at T_4_ compared to the control subjects was 22.9% (Fig 5).

RM-ANOVA revealed a significant variation in the LSIs for the acceleration impulse during take-off (LSI_CMJto_) (*F*_(3,33)_=6.33, *P*=0.01, $\eta_{p}^{2}$ = 0.37). The LSI_CMJto_ was significantly lower at T_2_ than at T_1_ (*T*(12)=2.21, *P*=0.05, *d*=0.50) and significantly higher at T_3_ than at T_2_ (*T*(11)=3.21, *P*=0.01, *d*=0.53) and at T_4_ than at T_3_ (*T*(17)=3.10, *P*=0.01, *d*=0.45). The ACL subjects had a significantly lower LSI_CMJto_ at T_4_ than the control subjects (*T*(38)=2.81, *P*=0.01, *d*=0.89). The deficit in the acceleration impulse during take-off in the injured leg compared to the uninjured leg was 41% at T_4_.

**Fig 6:**
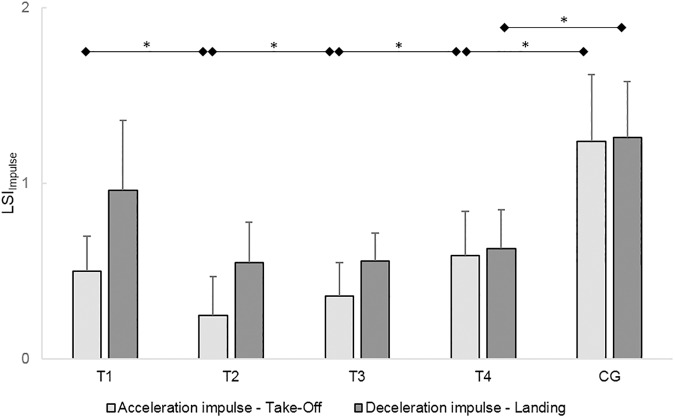
Leg symmetry indices (LSIs) of acceleration impulses during take-off and LSIs of deceleration impulses during landing of the counter movement jumps (CMJs). Mean LSIs and 95% confidence intervals of the acceleration and deceleration impulses of the CMJs. The acceleration impulses were measured during take-off and the deceleration impulses during landing of the ACL subjects (T_1_-T_4_) and the control subjects (CG). Test sessions with significant (*P*≤0.05) differences are marked with an asterisk (*).

RM-ANOVA revealed no significant variation of the LSIs of the deceleration impulse during landing (LSI_CMJla_) in the CMJs (*F*_(3,33)_=1.76, *P*=0.20, $\eta_{p}^{2}$ = 0.14). The LSI_CMJla_ of the ACL subjects was significantly lower at T_4_ than the LSI_CMJl_ the of the control subjects (*T*(38)=3.16, *P<*0.01, *d*=1.00). In the ACL subjects, the deceleration impulse during landing was 37% lower in the injured leg than in the uninjured leg at T_4_. (Fig 6)

Summarized, although the jumping height and the LSIs of the acceleration impulse during take-off increased up to six months post-reconstruction, the ACL subjects had not reached the level of the healthy controls in jumping height and the LSIs of the acceleration impulses during take-off and deceleration impulses during landing.

#### One-leg jumps (OLJ)

**Fig 7:**
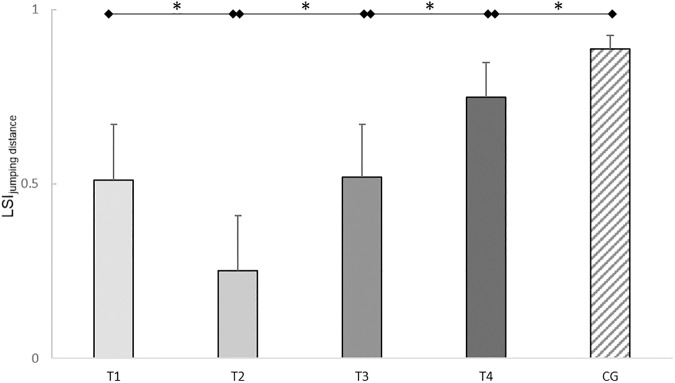
Leg symmetry indices (LSIs) of jumping distances of the one leg jumps (OLJs). Mean LSIs and 95% confidence intervals of the jumping distances of the OLJs of the ACL subjects (T_1_-T_4_) and the control subjects (CG). Test sessions with significant (*P*≤0.05) differences are marked with an asterisk (*).

RM-ANOVA revealed a significant variation of the LSIs of the jumping distances (*F*_(3,45)_=13.43, *P*<0.01, $\eta_{p}^{2}$ = 0.47). The LSIs of the jumping distance dropped from T_1_ to T_2_ (*T*(16)=3.32, *P*=0.01, *d*=0.78). From T_2_ to T_3_ (*T*(15)=3.56, *P*=0.01, *d*=0.79) and from T_3_ to T_4_ (*T*(16)=3.66, *P*<0.01, *d*=0.98) significant increases of the LSIs for jumping distance were detected. The LSI of the jumping distance was significantly lower in the ACL subjects at T_4_ compared to the control subjects (*T*(38)=2.50, *P*=0.02, *d*=0.79). In the ACL subjects, the jumping distance of the injured leg was 25.1% lower compared to the uninjured leg at T_4_ (Fig 7).

**Fig 8:**
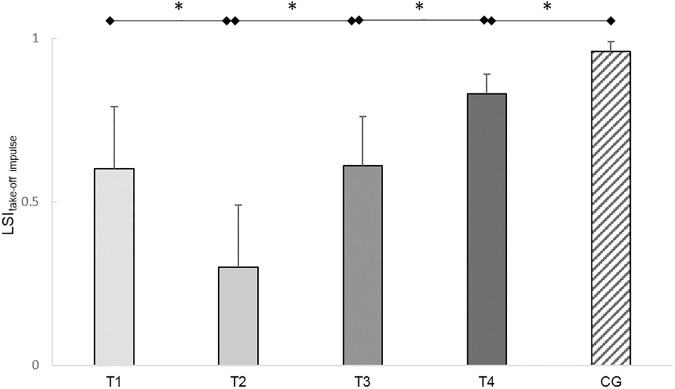
Leg symmetry indices (LSIs) of the acceleration impulses during take-off of the one leg jumps (OLJs). Mean LSIs and confidence intervals of the acceleration impulses during take-off of the OLJs of the ACL subjects (T_1_-T_4_) and the control subjects (CG). Test sessions with significant (*P*≤0.05) differences are marked with an asterisk (*).

RM-ANOVA revealed a significant variation in the LSI for the acceleration impulse during take-off in the ACL subjects (LSI_OLJto_) (*F*_(3,45)_=12.22, *P*<0.01, $\eta_{p}^{2}$ = 0.45). The LSIs of acceleration impulse dropped from T_1_ to T_2_ (*T*(16)=3.32, *P*<0.01, *d*=0.80). From T_2_ to T_3_ (*T*(15)=3.56, *P*<0.01, *d*=0.87) and from T_3_ to T_4_ (*T*(16)=3.66, *P*<0.01, *d*=0.99) significant increases of the LSIs of acceleration impulses were detected. However, the LSI_OLJto_ in the ACL subjects at T_4_ was significantly lower compared to the control subjects (*T*(38)=3.30, *P*<0.01, *d*=1.04). The acceleration impulse of the injured leg was 17% lower compared to the uninjured leg at T_4_ (Fig 8).

Summarized, the LSIs of the jumping distances and of the take-off impulses increased in the ACL subjects up to six months post-reconstruction, however, remained lower than the LSIs of the healthy control subjects.

#### Isometric force tests

**Fig 9:**
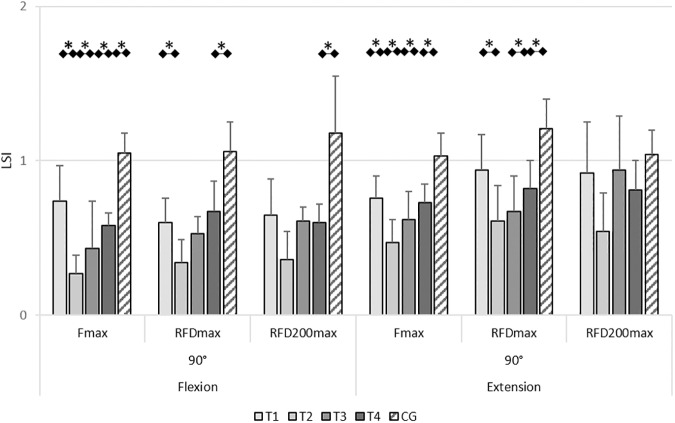
Leg symmetry indices (LSIs) of the isometric force parameters in 90° flexion and 90° extension condition. Exemplary results of mean LSIs 95% confidence intervals of the maximum force (F_max_), maximum rate of force development (RFD_max_) and maximum rate of force development of the initial 200ms of contraction (RFD_200max_) in 90° knee flexion and 90° knee extension conditions. Detailed results of the LSIs of all analyzed parameters and significant differences between of all parameters between the test sessions are given in S2 Table. Test sessions with significant (*P*≤0.05) differences are marked with an asterisk (*) in Fig 9 and are mentioned in the Results section of the manuscript.

The LSIs of F_max_ (LSI_Fmax_), RFD_max_ (LSI_RFDmax_) and RFD_200max_ (LSI_RFD200max_) are given in S2 Table. Therein, all conditions where the LSIs differed significantly are listed, including effect sizes of the post-hoc *t*-tests. Fig 9 shows exemplary results of the LSIs for F_max_, RFD_max_ and RFD_200max_ during knee flexion and knee extension at 90°. The results of the 110° condition showed similar trends.

RM-ANOVA revealed a significant variation in LSI_Fmax_ at 90° flexion (*F*_(3,45)_=12.11, *P*<0.01, $\eta_{p}^{2}$ = 0.45) and 110° flexion (*F*_(3,33)_=4.96, *P*<0.01, $\eta_{p}^{2}$ = 0.31) as well as 90° extension (*F*_(3,45)_=7.38, *P*<0.01, $\eta_{p}^{2}$ = 0.33) and 110° extension (*F*_(3,39)_=14.06, *P*< 0.01, $\eta_{p}^{2}$ = 0.52). The ACL subjects showed significantly lower values for LSI_Fmax_ in all flexion and extension conditions at T_2_ compared to T_1_. Except for 110° flexion from T_3_ to T_4_, all other flexion and extension conditions showed significant increases in the LSI_Fmax_ from T_2_ to T_3_ and from T_3_ to T_4_. The LSI_Fmax_ in the ACL subjects at T_4_ were significantly lower than those of the control subjects at 90° and 110° knee flexion as well as 90° and 110° knee extension. The deficit of F_max_ in the injured leg compared to the uninjured leg was between 25% (110° extension) and 51% (110° flexion) at T_4_.

RM-ANOVA revealed a significant variation in LSI_RFDmax_ in the ACL subjects at 90° flexion (*F*_(3,57)_=3.28, *P*=0.03, $\eta_{p}^{2}$ = 0.16) as well as at 90° extension (*F*_(3,57)_=3.28, *P*=0.01, $\eta_{p}^{2}$ = 0.29) and 110° extension (*F*_(3,51)_=4.45, *P*=0.01, $\eta_{p}^{2}$ = 0.21). The LSI_RFDmax_ was significantly lower in all tested conditions at T_2_ compared to T_1_ (S2 Table). At 110° and 90° knee extension, significantly higher LSI_RFDmax_ was found at T_4_ compared to T_3_. The LSI_RFDmax_ in the ACL subjects at T_4_ were significantly lower than those of the control subjects at 90° and 110° knee flexion as well as 90° and 110° knee extension. The deficit in RFD_max_ in the injured leg compared to the uninjured leg was between 18% (90° extension) and 44% (110° flexion) at T_4_.

RM-ANOVA revealed a significant variation in LSI_RFD200max_ at 110° knee flexion (*F*_(3,48)_=3.28, *P*=0.03, $\eta_{p}^{2}$ = 0.17) and 110° knee extension (*F*_(3,51)_=4.19, *P*=0.02, $\eta_{p}^{2}$ = 0.20). LSI_RFD200max_ was significantly lower at T_1_ compared to T_2_ as well as significant higher at T_4_ compared to T_3_. The LSI_RFD200max_ in the ACL subjects at T_4_ were significantly lower than those of the control subjects at 90° and 110° knee flexion as well as 90° and 110° knee extension (S2 Table). The deficit in RFD_200max_ in the injured leg compared to the uninjured leg was between 19% (90° extension) and 40% (90° flexion) at T_4_.

Summarized, the LSIs of all parameters of the isometric tests dropped from pre- to post-reconstruction time. Afterwards the LSIs increased in the knee flexion and extension conditions up to six months post-reconstruction. This was especially seen in the LSIs_Fmax_ over all testing conditions, but not in all testing conditions for LSIs_RFDmax_ and LSIs_RFD200max_. All LSIs of the analyzed strength parameters were lower six months after reconstruction compared to the healthy control subjects.

## Discussion

This was the first study investigating specific components, determining and limiting knee function, after ACL reconstruction. This was implemented by the combination of self-evaluating questionnaires, functional clinical tests as well as static and dynamic functional FPTs from pre- to six months post-reconstruction with four test sessions. With this study design a more detailed understanding of the course of the functional state of the knee during the rehabilitation process was enabled. On a macroscopic level this study revealed three main findings: Firstly, the LSIs decreased after the ACL tear and reconstruction, indicating that the injured leg loses functionality from pre- to post-reconstruction. Secondly, the LSIs increased from six weeks post-reconstruction up to six months post-reconstruction, and thirdly, the LSIs of the ACL group subjects remained lower compared to the LSIs of the control subjects at six months post-reconstruction.

The reduction of the LSIs from pre- to post-reconstruction was significant in almost all tested parameters. This primarily shows the influence of the ACL tear and reconstruction on joint function in clinical tests and FPTs as well as the impact of the ACL tear of the individuals’ QoL, which could be derived by the low self-evaluated knee function. Besides the low self-evaluated state, the low performance in the functional clinical tests and FPTs are not unexpected as the important role of the ACL for knee joint functionality is undeniably described [3, 6, 8]. The increase of functionality, according to the rising LSIs, in almost all parameters from six weeks post-reconstruction up to three and six months post-reconstruction shows that the analyzed rehabilitation programs enhance functionality in the reconstructed leg although the ACL group subjects did not reach the level of the control subjects in nearly all of the conducted tests. These results are discussed in details in the subsequent sections.

### Functional clinical tests

Despite the enhancement of the LSIs, they remained on a lower level in nearly all parameters at six months post-reconstruction compared to the healthy control group subjects. These lower LSIs were seen in the functional clinical tests and the FPTs. The reduced LSIs of the LC measurements at S15 show, that the thigh musculature was still atrophied in the ACL group. Such thigh atrophy was described before and can be explained by the traumatic rupture and the subsequent neuromuscular changes in the injured leg [10, 11, 14]. Additionally, the ACL subjects show reduced LSIs for passive ROM in knee extension and flexion compared to the control subjects six months post-reconstruction independently of the increasing LSIs in passive ROM over the four test sessions. Such knee ROM deficits in dynamic and static conditions were described previously [18, 23], as well as the importance of full ROM recovery, especially in knee flexion, for full knee joint recovery in dynamic movements [12, 30]. As both parameters have not recovered up to six months post-reconstruction, it is not surprising that the ACL group subjects show pronounced LSI deficiencies in the FPTs.

### One-legged and bilateral jumps

LSI deficiencies were apparent in the dynamic jumping FPTs compared to the control subjects, at six months post-reconstruction. Although the LSIs of jumping distances in the OLJs increased up to six months post-reconstruction, the ACL subjects showed pronounced LSI deficits for jumping distance compared to the control subjects. The ACL subjects could only realize a jumping distance with the injured leg of 74.9% of the uninjured leg. As it is described that a minimum of 85% should get reached before the performance of the reconstructed leg is declared normal [3, 5, 6, 15, 16, 19, 23, 24, 26–28, 41], the results of our study yielded remarkable deficits in one-legged jumping performance in the reconstructed leg and therewith no normal symmetry level of the ACL reconstructed subjects.

These one-legged movement deficits were underlined by the bilateral CMJs performance, where the jumping height was reduced by 23.9% compared to the control subjects. In contrast to unilateral OLJs for distance or height [3, 15, 16, 18, 19, 23, 24, 26–28, 41], bilateral CMJs are underrepresented in studies evaluating the functional outcomes after ACL tears. However, the evaluation of CMJs provides important information about the injured leg influences to the performance of bilateral movements. Especially, by the consideration of the acceleration impulse during take-off and the deceleration impulse during landing. These impulses provide general information about the ability to generate, apply and compensate for forces over a specific time in order to realize a specific task.

Although, the LSIs of the impulse parameters of the ACL subjects also improved over time, the LSIs of the acceleration impulse during take-off and the deceleration impulse during landing were lower than the LSIs of the control subjects at six months post- reconstruction, indicating a clear asymmetrical loading pattern. This asymmetrical load pattern was seen as a 41% lower acceleration impulse during take-off in the injured leg compared to the uninjured leg. This demonstrates a shift of load generation to the uninjured leg during take-off. This results also in a reduced overall take-off impulse, which explains the reduced jumping heights in the CMJs. During bilateral landing of the CMJs the deceleration impulse in the injured leg was 37% lower than in the uninjured leg, implying as well a shift of load compensation to the uninjured leg. Surprisingly, in the OLJs, the ACL subjects showed only a 17% deficit in the acceleration impulse during take-off in the injured compared to the uninjured leg. This deficit in acceleration impulse during take-off was lower in the OLJs than in the CMJs. This demonstrates that during take-off in bilateral CMJs, the ACL subjects shifted more load to their uninjured leg than the relative leg deficit was in the unilateral OLJs.

Collectively, the results of these parameters lead to the conclusion that besides deficits between the legs in the functional clinical tests in dynamic performance remarkable deficiencies, especially in bilateral jumping, in the injured leg compared to the uninjured leg at six months post-reconstruction exist. Similar compensation strategies involving the uninjured leg in jumps have been described in OLJs before, but not in CMJs [18, 23]. The results implicate that for comprehensive evaluation and monitoring of knee joint functionality one leg movement tasks should be supplemented by bilateral movement tasks, such as CMJs. The results of the functional clinical tests and the FPTs demonstrate how essential comprehensive test batteries are, including clinical tests and FPTs, for determining leg deficiencies more graduate and for providing a comprehensive state of the knee functionality.

### Isometric force tests

The deficiencies in the reconstructed leg in the jumping tasks are underlined by deficiencies of the reconstructed leg in the isometric force tests. Herein, the LSIs improved from about six weeks post-reconstruction up to six months post-reconstruction. However, the LSIs of the ACL subjects were reduced compared to the control subjects’ LSIs in F_max_, RFD_max_ and RFD_200max_ at six months post-reconstruction.

RFD_200max_ is important for the rehabilitation process evaluation because during movements such as postural balance corrections in everyday life or jumping in intense sports, contraction times of up to 200ms are required. These contraction times are shorter than the time normally needed to reach maximal isometric force, which is between 300 and 500ms [11, 13].

The developments of F_max_ in comparison to RFD_max_ and RFD_200max_ indicate that neuronal adaptation processes recover on a higher level in comparison to adaptations of the legs’ muscle volume up to six months post-reconstruction. In flexion and extension condition, F_max_ shows a stepwise increase of the leg strength with every test session, without reaching the level of the control group at six months post-reconstruction. Especially in knee extension, the RFD does not show such a time effect. In particular in RFD_200max_ there is no difference in the ACL group compared to the control group in test sessions three and four. As RFD is in general strongly related to efferent neuronal capacities, it appears that the RFD deficits are not that pronounced than the deficits in maximum force generation [13]. In contrast, the maximum force, which is substantially reduced in the ACL group compared to the healthy control group, is strongly related to the muscle volume. This result is in accordance to the analyses of the LCs. It was found, that at the fourth test session the circumference of the thigh in the area of the biggest muscle belly (S15) stayed reduced in the injured leg compared to the non-injured leg and additionally the relative circumference of the injured leg in the ACL group was reduced compared to the control subjects.

Deficits between the ACL subjects and the control subjects six months post-reconstruction were observed under knee flexion and extension conditions and at knee angles of 90° and 110°. The injured leg deficits compared to the uninjured leg of the ACL group subjects were between 25% (110° extension) to 51% (110° flexion) in F_max_, between 18% (90° extension) to 44% (110° flexion) in RFD_max_, and between 19% (90° extension) to 40% (90° flexion) in RFD_200max_. These deficiencies are higher than those reported in the literature [5].

The deficits in comparison to the control group could be explained by a deficiency of the hamstrings muscles, which could be caused by the graft removal of tendons of hamstrings muscles. This was underlined by the more prominent deficiency in the injured leg during flexion than during extension. Thus, the deficient passive ROM during flexion was associated with deficiencies in isolated flexion force generation in the injured leg along with deficiencies in the FPTs. Due to the importance of flexion capabilities in dynamic performance tasks and the agonistic function of the hamstrings to the ACL [12, 30], it appears that these limitations in ROM in knee flexion and in generating forces could be an explanation for the shift of load to the uninjured side in bilateral CMJs and the performance discrepancy in the unilateral OLJs [18, 23] and the generally reduced functionality compared to the control group subjects even at six months post-reconstruction.

### Limitations

The sample consisted of subjects of both genders with a wide range of age and different pre-injury activity levels. Additionally, depending on the functional state, the subjects could perform activities beyond institutional therapeutical rehabilitation to a variable extent. The ability to perform autonomous therapeutic-independent training is strongly associated with the functional status and the intrinsic motivation of ACL reconstructed individuals. Higher training loads typically result in a higher functional state, due to the fact that the structures determining functionality, get positively influenced by an increased amount of training. Depending on the purposes, these issues need to be controlled in future studies. Due to the reason that this study aimed to draw a general picture of the functional outcome after ACL reconstruction we did not restrict the inclusion criteria of the sample in relation to the mentioned criteria. Nonetheless, more homogenous samples could lead to more specific results in relation to the drawn sample.

### Practical implications

The results of this study imply that detailed analyses of specific components, determining and limiting knee function, monitored repetitively after ACL reconstruction, improves the understanding of the recovery process of knee functionality. Therefore, the applied test battery enables clinicians and therapists to detect functionality very detailed, which provides a quantitative base for adapting the rehabilitation program more individually in relation to the respective individual functional state. This helps to achieve the best rehabilitative outcome of the ACL reconstructed individuals. In contrast, functional performance testing at one specific time point after reconstruction, as well as placing reliance only on functional clinical testing or the time period after reconstruction seems not adequate for determining functionality of ACL reconstructed individuals [34]. Moreover, the results of this study show that clinicians and therapists have to be aware of limited restoration of knee functionality of ACL reconstructed subjects in comparison to healthy control subjects up to six months after reconstruction. Therefore, caution is advised before individuals get released in pre-injury sports and further training recommendations are essential.

### Conclusions

Summarized it can be stated that functionality of the ACL reconstructed subjects follows a uniform course, with a decrease from immediately pre-reconstruction time to six weeks post-reconstruction and a subsequent increase of functionality up to three and six months post-reconstruction. This shows that the applied common rehabilitation program enhances knee joint functionality up to six months post-reconstruction. However, at six months post-reconstruction the ACL reconstructed subjects have not reached the functional state of healthy control subjects in hardly any parameter, not even in their self-evaluated functional knee state and their self-determined activity level.

Accordingly, our general hypothesis was confirmed, namely, that the functionality of the ACL reconstructed subjects of this study could not be called ‘normal’ from subjective and objective viewpoints at six months post-reconstruction.

## Supporting information

S1 Table Summarized rehabilitation program of the ACL reconstructed subjects. Summarized rehabilitation programs and performed recreational and/or sports activities of the ACL reconstructed subjects up to 6 months post-ACL reconstruction. Distinguished in physiotherapeutic exercises (PT), activities of daily living (ADL), and recreational or sports activities (SP).
(DOCX)

S2 Table Results of the isometric force tests. Leg symmetry indices with 95% confidence intervals of the parameters analyzed in the isometric force tests: Peak force (F_max_), maximum rate of force development (RFD_max_) and maximum rate of force development in the first 200ms after contraction initiation (RFD_200max_) standardized by body weight (kg). In the last column all significant results of the post-hoc analysis are illustrated (*P* < 0.05).
(DOCX)
