# Supplementary material for: How does functionality proceed in ACL reconstructed subjects? Proceeding of functional performance from pre- to six months post-ACL reconstruction
Source: PLoS One. 2017 May 31;12(5):e0178430. doi: 10.1371/journal.pone.0178430 (PMC5451139; doi:10.1371/journal.pone.0178430)
Supplement: S1 Table — Summarized rehabilitation programs and performed recreational and/or sports activities of the ACL reconstructed subjects up to 6 months post-ACL reconstruction. Distinguished in physiotherapeutic exercises (PT), activities of daily living (ADL), and recreational or sports activities (SP). (DOCX) [file pone.0178430.s001.docx]

**S1 Table. Summarized rehabilitation program of the ACL reconstructed subjects.**

|  | **Time post-reconstruction** | **Rehabilitation exercises** |
| --- | --- | --- |
| **1^st^ stage** | 1^st^ week | PT: Lymphatic drainage, physical therapy (passive ROM exercises, massage). |
|  |  | ADL: Walking with crutches. |
|  | 2^nd^ week | PT: Lymphatic drainage, physical therapy (passive ROM exercises, massage, closed-kinetic chain exercises). |
|  |  | ADL: Walking with crutches. |
|  | 3^rd^ week | PT: Lymphatic drainage, physical therapy (passive ROM exercises, massage, closed-kinetic chain exercises). |
|  |  | ADL: Walking with crutches. |
|  | 4^th^ week | PT: Lymphatic drainage, physical therapy (passive ROM exercises, massage, closed-kinetic chain exercises, stability exercises). |
|  |  | ADL: Walking without or with one crutch. |
|  | 5^th^ week | PT: Proprioceptive training (One-legged stance, step-up forward/ backward, stability exercises), ROM exercises, closed-kinetic chain exercises. |
|  |  | ADL: Walking without or with one crutch, stair climbing. |
|  | 6^th^ week | PT: Proprioceptive training (One-legged stance, step-up forward/ backward, stability exercises), ROM exercises, closed-kinetic chain exercises. |
|  |  | ADL: Walking without crutches, stair climbing ergometer cycling, Aqua jogging. |
|  | 7^th^ week | PT: Proprioceptive training (One-legged stance, step-up forward/ backward, stability exercises), ROM exercises, closed-kinetic chain exercises. |
|  |  | ADL: Walking without crutches, ergometer cycling, Aqua jogging. |
| **2^nd^ stage** | 8^th^ week | PT: core strength training, proprioceptive training unstable surface, gymnastics/stretching. |
|  |  | ADL: Walking, (ergometer) cycling, Aqua jogging. |
|  | 9^th^ week | PT: core strength training, proprioceptive training unstable surface, gymnastics/stretching. |
|  |  | ADL: Walking, (ergometer) cycling. |
|  | 10^th^ week | PT: core strength training, low-intensity lunges, leg press, proprioceptive training unstable surface, gymnastics/stretching. |
|  |  | ADL: Walking, (ergometer) cycling. |
|  | 11^th^ week | PT: core strength training, medium-intensity lunges, leg press, proprioceptive training unstable surface. |
|  |  | ADL: (Ergometer) cycling, Cross-Trainer, Walking on treadmill. |
|  | 12^th^ week | PT: core strength training, medium-intensity lunges, leg press, proprioceptive training unstable surface. |
|  |  | ADL: (Ergometer) cycling, Cross-Trainer, Walking on treadmill. |
|  | 13^th^ week | PT: Core strength training (leg press, Abduction, knee flexion), proprioceptive training unstable surface, one-legged lunges. |
|  |  | ADL: Cross-Trainer, Walking on treadmill, cycling. |
|  | 14^th^ week | ADL: Cross-Trainer, Walking on treadmill, cycling. |
| **3^rd^ stage** | 15^th^ week | PT: Core strength training (leg press, Abduction, knee flexion), proprioceptive training unstable surface. |
|  |  | SP: Swimming, cycling. |
|  | 16^th^ week | PT: Core strength training (leg press, Abduction, knee flexion), proprioceptive training unstable surface |
|  |  | SP: Swimming, cycling. |
|  | 17^th^ week | PT: Core strength training (leg press, Abduction, knee flexion), proprioceptive training unstable surface |
|  |  | SP: Swimming, cycling, moderate jogging. |
|  | 18^th^ week | PT: Core strength training (leg press, Abduction, knee flexion), proprioceptive training unstable surface |
|  |  | SP: Swimming, cycling, moderate jogging. |
|  | 19^th^ week | PT: Core strength training (leg press, Abduction, knee flexion), proprioceptive training unstable surface |
|  |  | SP: Swimming, cycling, moderate jogging. |
|  | 20^th^ week | PT: Core strength training (leg press, Abduction, knee flexion), proprioceptive training unstable surface |
|  |  | SP: Swimming, cycling, moderate jogging. |
|  | 21^st^ week | PT: Core strength training (leg press, Abduction, knee flexion), proprioceptive training unstable surface |
|  |  | SP: Swimming, cycling, moderate jogging. |
|  | 22^nd^ week | PT: Core strength training (leg press, Abduction, knee flexion), proprioceptive training unstable surface |
|  |  | SP: One-legged jumps for distance and vertical, swimming, cycling. |
|  | 23^rd^ week | PT: Core strength training (leg press, Abduction, knee flexion), proprioceptive training unstable surface |
|  |  | SP: unilateral and bilateral lateral jumps, jogging. |
|  | 24^th^ week | PT: Core strength training (leg press, Abduction, knee flexion), proprioceptive training unstable surface |
|  |  | SP: jogging, pre-injury sports. |
|  | 25^th^ week | PT: Core strength training (leg press, Abduction, knee flexion), proprioceptive training unstable surface |
|  |  | SP: jogging, pre-injury sports |
|  | 26^th^ week | PT: Core strength training (leg press, Abduction, knee flexion), proprioceptive training unstable surface |
|  |  | SP: jogging, pre-injury sports |
