# Supplementary material for: How does functionality proceed in ACL reconstructed subjects? Proceeding of functional performance from pre- to six months post-ACL reconstruction
Source: PLoS One. 2017 May 31;12(5):e0178430. doi: 10.1371/journal.pone.0178430 (PMC5451139; doi:10.1371/journal.pone.0178430)
Supplement: S2 Table — Leg symmetry indices with 95% confidence intervals of the parameters analyzed in the isometric force tests: Peak force (Fmax), maximum rate of force development (RFDmax) and maximum rate of force development in the first 200ms after contraction initiation (RFD200max) standardized by body weight (kg). In the last column all significant results of the post-hoc analysis are illustrated (P < 0.05). (DOCX) [file pone.0178430.s002.docx]

**S2 Table. Results of the isometric force tests**

| **Parameter** | **Test condition** | **T_1_** | **T_2_** | **T_3_** | **T_4_** | **Control group** | **Significant differences** |
| --- | --- | --- | --- | --- | --- | --- | --- |
| **F_max_**  **[N/kg]** | Flexion 90° | 0.74 ± 0.22 | 0.27 ± 0.10 | 0.43 ± 0.09 | 0.58 ± 0.08 | 1.05 ± 0.13 | T_1_/T_2_: $T\left( 17 \right)=5.00, P<0.01, d=0.91$ |
|  |  |  |  |  |  |  | T_2_/T_3_: $T\left( 16 \right)=3.22, P=0.01, d=0.82$ |
|  |  |  |  |  |  |  | T_3_/T_4_: $T\left( 17 \right)=3.28, P<0.01, d=0.77$ |
|  |  |  |  |  |  |  | T_4_/CG: $T\left( 38 \right)=6.05, P<0.01, d=1.91$ |
|  | Flexion 110° | 0.64 ± 0.18 | 0.34 ± 0.20 | 0.46 ± 0.13 | 0.49 ± 0.09 | 1.02 ± 0.12 | T_1_/T_2_: $T\left( 12 \right)=2.89, P=0.01, d=1.10$ |
|  |  |  |  |  |  |  | T_2_/T_3_: $T\left( 11 \right)=4.62, P<0.01, d=0.54$ |
|  |  |  |  |  |  |  | T_3_/T_4_: $T\left( 15 \right)=2.47, P=0.03, d=0.38$ |
|  |  |  |  |  |  |  | T_4_/CG: $T\left( 38 \right)=7.00, P<0.01, d=2.21$ |
|  | Extension 90° | 0.76 ± 0.13 | 0.47 ± 0.13 | 0.62 ± 0.15 | 0.73 ± 0.12 | 1.03 ± 0.15 | T_1_/T_2_: $T\left( 17 \right)=4.66, P<0.01, d=0.88$ |
|  |  |  |  |  |  |  | T_2_/T_3_: $T\left( 16 \right)=3.45, P=0.01, d=0.54$ |
|  |  |  |  |  |  |  | T_4_/CG: $T\left( 38 \right)=3.06, P<0.01; d=0.97$ |
|  | Extension 110° | 0.83 ± 0.14 | 0.46 ± 0.18 | 0.63 ± 0.10 | 0.75 ± 0.09 | 1.08 ± 0.09 | T_1_/T_2_: $T\left( 14 \right)=4.98, P<0.01, d=1.14$ |
|  |  |  |  |  |  |  | T_2_/T_3_: $T\left( 13 \right)=3.54, P< 0.01, d=0.61$ |
|  |  |  |  |  |  |  | T_3_/T_4_: $T\left( 16 \right)=4.57, P<0.01, d=0.80$ |
|  |  |  |  |  |  |  | T_4_/CG: $T\left( 38 \right)=5.02, P<0.01; d=1.59$ |

| **RFD_max_**  **[N/kg*s]** | Flexion 90° | 0.60 ± 0.16 | 0.34 ± 0.13 | 0.53 ± 0.11 | 0.67 ± 0.20 | 1.06 ± 0.19 | T_1_/T_2_: $T\left( 19 \right)=2.97, P<0.01, d=0.75$ |
| --- | --- | --- | --- | --- | --- | --- | --- |
|  |  |  |  |  |  |  | T_4_/CG: $T\left( 38 \right)=2.85, P<0.01, d=0.90$ |
|  | Flexion 110° | 0.59 ± 0.18 | 0.52 ± 0.26 | 0.40 ± 0.14 | 0.56 ± 0.11 | 1.04 ± 0.17 | T_1_/T_2_: $T\left( 16 \right)=2.58, P=0.02, d=0.54$ |
|  |  |  |  |  |  |  | T_3_/T_4_: $T\left( 19 \right)=3.79, P<0.01, d=0.69$ |
|  |  |  |  |  |  |  | T_4_/CG: $T\left( 38 \right)=4.66, P<0.01, d=1.47$ |
|  | Extension 90° | 0.94 ± 0.22 | 0.61 ± 0.20 | 0.67 ± 0.18 | 0.82 ± 0.18 | 1.21 ± 0.19 | T_1_/T_2_: $T\left( 19 \right)=2.60, P=0.02, d=0.62$ |
|  |  |  |  |  |  |  | T_3_/T_4_: $T\left( 19 \right)=3.15, P<0.01, d=1.06$ |
|  | Extension 110° | 0.89 ± 0.26 | 0.55 ± 0.23 | 0.56 ± 0.19 | 0.69 ± 0.14 | 1.12 ± 0.15 | T_1_/T_2_: $T\left( 17 \right)=2.69, P=0.02, d=0.72$ |
|  |  |  |  |  |  |  | T_3_/T_4_: $T\left( 19 \right)=2.49, P=0.02, d=0.58$ |
|  |  |  |  |  |  |  | T_4_/CG: $T\left( 38 \right)=4.22, P<0.01; d=1.33$ |
| **RFD_200max_**  **[N/kg*s]** | Flexion 90° | 0.65 ± 0.23 | 0.36 ± 0.16 | 0.61 ± 0.30 | 0.60 ± 0.12 | 1.18 ± 0.37 | T_4_/CG: $T\left( 38 \right)=2.93, P<0.01, d=0.93$ |
|  | Flexion 110° | 0.61 ± 0.18 | 0.44 ± 0.23 | 0.44 ± 0.14 | 0.61 ± 0.22 | 1.12 ± 0.40 | T_1_/T_2_: $T\left( 16 \right)=2.35, P=0.03, d=0.54$ |
|  |  |  |  |  |  |  | T_4_/CG: $T\left( 38 \right)=2.21, P<0.03, d=0.70$ |
|  | Extension 90° | 0.92 ± 0.32 | 0.54 ± 0.22 | 0.94 ± 0.34 | 0.81 ± 0.19 | 1.04 ± 0.16 |  |
|  | Extension 110° | 0.93 ± 0.30 | 0.42 ± 0.20 | 0.54 ± 0.20 | 0.66 ± 0.18 | 1.22 ± 0.28 | T_1_/T_2_: $T\left( 17 \right)=3.01, P<0.01, d=0.90$ |
|  |  |  |  |  |  |  | T_4_/CG: $T\left( 38 \right)=3.23, P<0.01, d=1.03$ |

Leg symmetry indices with 95% confidence intervals of the parameters analyzed in the isometric force tests: Peak force (F_max_), maximum rate of force development (RFD_max_) and maximum rate of force development in the first 200ms after contraction initiation (RFD_200max_) standardized by body weight (kg). In the last column all significant results of the post-hoc analysis are illustrated (*P* < 0.05).
